# Magnetic carbon Fe_3_O_4_ nanocomposites synthesized via Magnetic Induction Heating

**DOI:** 10.1038/s41598-023-34387-2

**Published:** 2023-05-04

**Authors:** L. Cervera-Gabalda, C. Gómez-Polo

**Affiliations:** 1 Departamento de Ciencias, Universidad Pública de Navarra, Campus de Arrosadia, 31006 Pamplona, Spain; 2 Institute for Advanced Materials and Mathematics (INAMAT2), Universidad Pública de Navarra, Campus de Arrosadia, 31006 Pamplona, Spain

**Keywords:** Materials science, Nanoscale materials, Applied physics

## Abstract

Magnetic Induction Heating (*MIH*) of magnetite nanoparticles is employed as a novel synthesis procedure of carbon based magnetic nanocomposites. Magnetic nanoparticles (Fe_3_O_4_) and fructose (1:2 weight ratio) were mechanically mixed and submitted to a *RF* magnetic field (305 kHz). The heat generated by the nanoparticles leads to the decomposition of the sugar and to the formation of an amorphous carbon matrix. Two sets of nanoparticles, with mean diameter sizes of 20 and 100 nm, are comparatively analysed. Structural (X-ray diffraction, Raman spectroscopy, Transmission Electron Microscopy (TEM)), electrical and magnetic (resistivity, SQUID magnetometry) characterizations confirm the nanoparticle carbon coating through the *MIH* procedure. The percentage of the carbonaceous fraction is suitably increased controlling the magnetic heating capacity of the magnetic nanoparticles. The procedure enables the synthesis of multifunctional nanocomposites with optimized properties to be applied in different technological fields. Particularly, Cr (VI) removal from aqueous media is presented employing the carbon nanocomposite with 20 nm Fe_3_O_4_ nanoparticles.

## Introduction

Nanocomposites, defined as multiphase materials where the matrix has at least one of its dimensions below 100 nm, represent a class of nanomaterials that has been extensively studied for several decades^1^. In particular, their multifunctional nature offers the possibility of applying them in different sectors covering a wide range of technological applications. The combination of different elements with specific catalytic, magnetic, electronic, and optical properties as well as surface functionality leads to their outstanding optimized performances. Among these systems, magnetic carbon nanocomposites stand out^2–6^, where the coating of magnetic nanoparticles with carbon, besides providing the desired multifunctionally, enhances thermal and chemical stability, oxidation resistance, ensuring biocompatibility and high specific surface area. A proper control of the functional groups on the carbon surface leads to highly efficient pollutant adsorbents, nanocarriers for drug delivery and cancer therapies, and optimizes the nanocomposite performance as electrocatalysts or energy storage components in lithium batteries. Additionally, the magnetic core enlarges the nanocomposite functionality: magnetic separation (recovery and recycling of pollutant adsorbents), biomedical applications (temperature assisted drug delivery, magnetic hyperthermia, image contrast agents) or microwave electromagnetic absorbents and filters, among others.

Different chemical processes can be employed in the preparation of carbon based nanoestructures^7^, most of them based on the thermal treatment of selected precursors at elevated temperatures (i.e. hydrothermal/solvothermal method, pyrolysis procedure, sol–gel process). However, non-thermal radiation heating procedures have been also explored and analysed as efficient techniques to synthesized nanomaterials and nanocomposites^8^. In these techniques (i.e. microwave heating, laser heating, Joule heating or Magnetic Induction Heating) the heat is directly and locally generated in the interior of objects, contrary to traditional thermal treatments where the external heat is transferred to objects via a media.

Particularly, Magnetic Induction Heating (*MIH*) is a traditional metallurgical tool based in the heating linked to the generation of eddy currents upon the application of an *AC* magnetic field in metallic (conductive) elements^9^. Joule heating gives rise to almost instantly heating at very high temperatures within seconds. However, it is well known and extensively reported within the last decades that magnetic nanoparticles (MNPs) can act as nanoheaters, linked to their magnetic hysteresis, relaxation and resonance processes when submitted to a *RF* magnetic field (magnetic hyperthermia)^10,11^. During the last decades, a great effort has been made in its application in the biomedical sector (drug delivery and cancer therapies)^12,13^. However, its application in other technological fields, such as new nanocomposite synthesis, has been scarcely analysed in the literature. Particularly, the heat generation of MNPs under *AC* magnetic field can be employed to calcine a metal oxide precursor gel^14^, controlled growth of metal–organic frameworks^15^, Ru hydrogenation nanocatalyst^16^ or optimized magnetic iron oxide nanoparticles^17^.

Regarding the synthesis of magnetic carbon nanocomposites, several methods have been reported, among them thermal decomposition methods stand out due to its simplicity, low-cost reactants, and the possibility to use different types of carbon sources^18,19^. In these synthesis procedures, usually an Fe source is employed playing the double role of catalyzing the carbon reduction and simultaneously form the magnetic nucleus.

In this work, we report the synthesis procedure of magnetic carbon nanocomposites obtained employing Fe_3_O_4_ magnetic nanoparticles as nanoheaters for the thermal decomposition of fructose. This sugar was selected as carbon source due to their simple thermal decomposition and employed as model for other carbon sources to obtain magnetic composites. The nanoparticles were mechanically mixed with the sugar and the mixture submitted to the action of a 305 kHz *AC* magnetic field. The heat generated by the nanoparticles thermally decomposes the sugar, giving rise to an amorphous electrically conductive carbon matrix, whose relative fraction depends on the power heating characteristics of the magnetic nanoparticles. The application as Cr (VI) nanoadsorbents in aqueous media is finally outlined.

## Results and discussion

**Figure 1:**
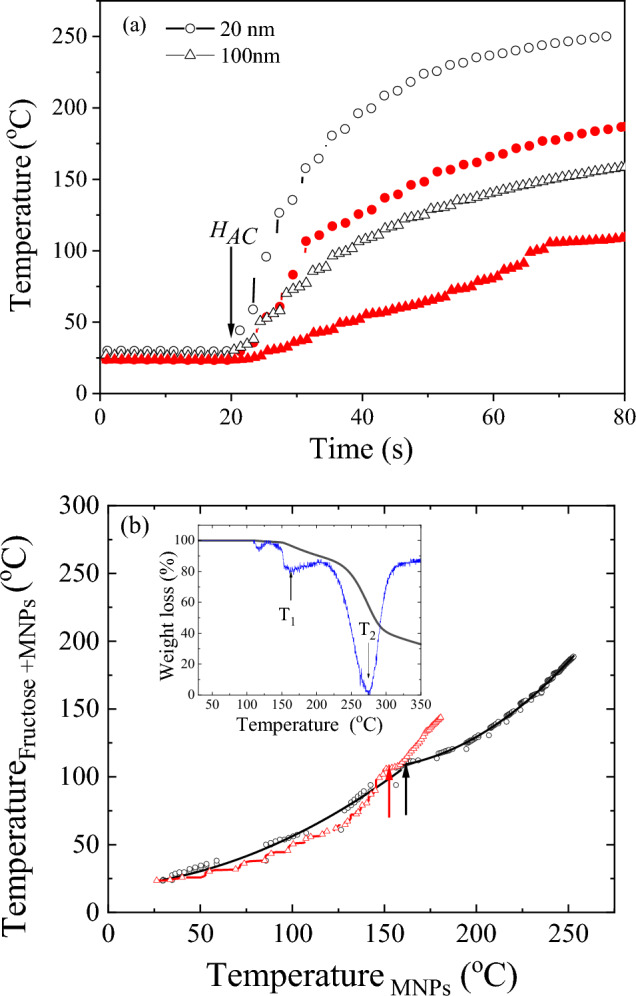
(**a**) *MIH *(Temperature versus time) curves for initial MNPs (open symbols) and fructose + MNPs (close symbols): (○) 20-MNP, (∆) 100-MNP. (**b**) Temperature for Fructose + MNPs versus the temperature of the initial MNPs under equivalent experimental conditions. The arrows mark the change of trend that should be linked to the melting point of fructose. Inset: TGA scan for the fructose, without nanoparticles (in blue the derivative curve in a.u.).

Two sets of Fe_3_O_4_ magnetic nanoparticles were employed, characterized by mean sizes around 20 and 100 nm (20-MNP and 100-MNP in the following). In order to check the heating capacity of initial MNPs, the increase of temperature for 20-MNP and 100-MNP under the effect of the (*RF*) *AC* magnetic field, *H*_*AC*_, (*f* = 305 kHz, amplitude 300 Oe) was first analysed as a function of the exposure time, *t*. Figure 1a shows the increase of temperature versus *t* for the initial nanoparticles. As it can be seen, the application of *H*_*AC*_ promotes a remarkable increase in temperature for short times, being higher for 20-MNP. For comparison, the temperature of the mixed samples (Fructose + MNPs) is also displayed under similar experimental conditions. When the MNPs are mixed with fructose, a smoother temperature increase is detected, being again remarkable lower the temperature increase when employing 100-MNPs. As will be discussed later, the lower heating capacity of 100-MNP should be ascribed to their largest nanoparticle size within the multidomain regime^20,21^. The occurrence of a non-linear temperature response in Fig. 1a for the analysed samples indicates the non-adiabatic nature of the employed set-up and the heat transfer of the system with the environment.

A clear change in the shape of the temperature curves is detected when the MNPs are mixed with fructose. While the curves for the initial MNPs can be properly fitted employing a 3nd order polynomial, a different trend occurs in the heating curves of the mixture. To analyse in further detail the thermal changes in the fructose as the temperature increases, Fig. 1b displays the measured temperatures for the Fructose + MNPs mixture and the temperature reached in the initial MNPs for the same exposure time. A kink (change on trend) can be clearly visualized in both mixtures for temperatures of the MNPs around 150 °C (arrows in Fig. 1b). Checking the decomposition process of fructose through TGA analysis (see inset of Fig. 1b), this point (*T*_*1*_) can be correlated with its melting temperature, being its final decomposition temperature reported around 270 °C (*T*_*2*_)^21^.

Accordingly, Magnetic Induction Heating (*MIH*) was explored as carbon coating procedure, where the MNPs would act as nanoheaters for the sugar thermal decomposition. A mixture of 200 mg of MNPs and 400 mg of fructose was mechanically mixed and submitted to the *AC* magnetic field (305 kHz). In these experiments, the amplitude of the *AC* magnetic field was adjusted to reach 200 °C at a similar heating rate (≈ 25 °C/min) in both samples. After 2 h of exposure time, the samples were cooled down to room temperature, properly washed and magnetically filtered (see “Methods” section for further details).

The *XRD* patterns were analysed for the initial MNPs and the *AC*-treated samples after being submitted to the *MIH* treatment. All the *XRD* patterns (see Fig. 1S, supplementary information) show the occurrence of a single Fe_3_O_4_ phase, confirmed through the Rietveld refinement (*Fd3m* space group). Moreover, the calculated cell parameter of the magnetite phase does not significantly change (*a* = *b* = *c* ≈ 8.3862 (11)) after the *MIH* procedure, displaying similar values than those of the bulk magnetite phase^22^. Moreover, the crystallite sizes (estimated through the Scherrer formula) remain nearly constant before and after the *MIH* treatment (<*d*> ≈ 18 nm and <*d*> ≈ 83 nm for 20-MNP and 100-MNP samples, respectively). It should be noted that the graphitic carbon peak in the treated samples is not clearly visible in the *XRD* diffractograms. This result would reflect the amorphous disordered nature of the carbon phase, as will be confirmed by Raman spectroscopy.

**Figure 2:**
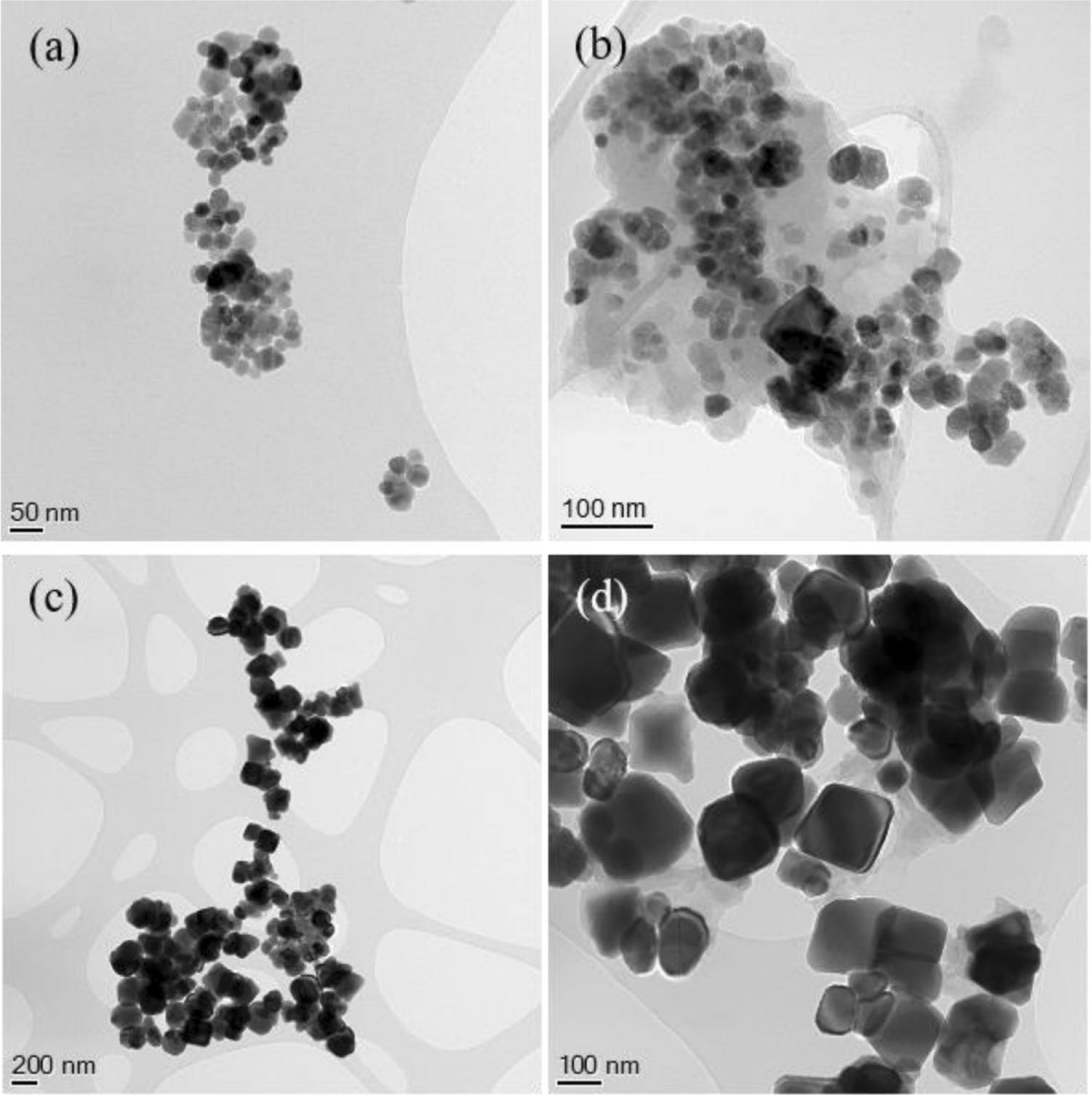
TEM images of 20-MNPs (**a**) initial and (**b**) *AC*-treated, and 100-MNPs sample, (**c**) initial and (**d**) *AC*-treated.

Figure 2 shows the TEM images of the initial and *AC*-treated MNPs. From the images, it can be concluded that after the *MIH* treatment a matrix is clearly visible for 20-MNP, although the carbon coating for the treated 100-MNP is rather inhomogeneous. Taking into account the previous magnetic induction heating characterization (see Fig. 1a), this result can be interpreted as a consequence of the lower heating capacity for the larger MNPs.

The MNPs size distribution (histograms) of the initial and *AC*-treated MNPs is depicted in Fig. S2, where the lognormal distribution function (solid line in the figures) characterizes the histograms. No remarkable changes can be detected in the MNPs size distributions after the *MIH* treatment. The calculated average particle diameter are: 27.7 ± 0.5 nm and 24.7 ± 0.3 nm (initial and *AC*-treated 20-MNPs) and 139 ± 3 nm and 137 ± 3 nm (initial and treated 100-MNP). In both cases, the values are larger than the crystalline size of the nanoparticles estimated from *XRD*. These differences, greater in the case of 100-MNP, are mainly correlated to the fact that the TEM sizes are measured within the visible grain limits, while the X-ray estimations provide the crystalline domain. Anyway, the estimated sizes for both MNPs explain the differences in their heating capacity. While most of the 20-MNPs will be in the monodomain regime, the multidomain state would dominate the magnetic state for 100-MNP. Notice that the reported critical size for single domain in magnetite nanoparticles is around 85 nm, depending this value on the nanoparticle shape^20^, being the maximum induction heating capacities ascribed to the monodomain state^23^.

**Figure 3:**
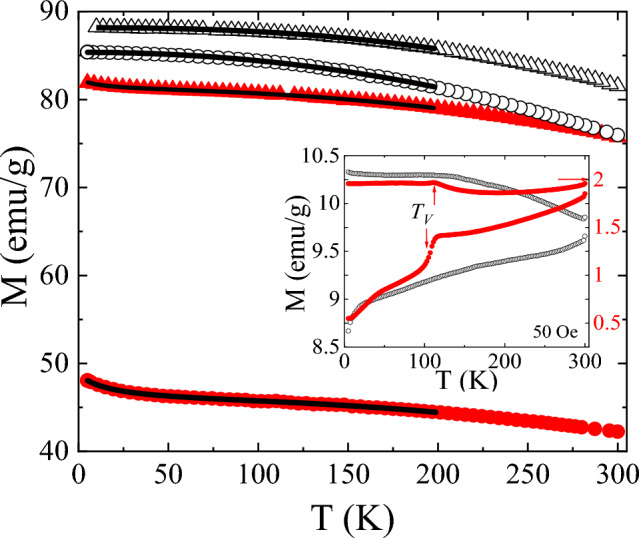
Temperature dependence of high field magnetization, *M*, (µ_0_*H* = 6 T) for initial MNPs (open symbols) and *AC*- treated samples (close symbols): (○) 20-MNP, (∆) 100-MNP. Inset: *ZFC–FC* magnetization curves for the 20-MNP samples (applied field 50 Oe).

The magnetic characterization confirms the previous structural analysis. Figure 3 shows the temperature dependence of the high field magnetization, *M*, under an applied magnetic field *µ*_*0*_H = 6 T. A reduction in *M* is observed for the treated MNPs in comparison with the initial nanoparticles. This reduction should be linked to the carbon content present in the treated MNPs since the magnetization values are calculated by dividing the measured magnetization by the total mass of the measured sample. Assuming a negligible magnetic contribution of the carbon phase, a high amount of carbon present in the *AC*-treated 20-MNP leads to a remarkable reduction in the measured magnetization. The sharper decrease of *M* for *T* < 50 K in the treated samples, would be indicative of the occurrence of additional magnetic phases (i.e. antiferromagnetic) as reported in other Fe–C nanocomposites obtained from the decomposition of sugars^21^.

In the current *MIH* synthesis procedure, the occurrence of this antiferromagnetic phase should be assumed to lie on the surface of the MNPs as a consequence of the interaction between the Fe cations on the nanoparticle surface and the melted fructose during the *MIH* procedure.

The low temperature magnetization (*T* < 200 K) was fitted to the Bloch law, where a Curie–Weiss contribution was included in the *AC-*treated samples: $M\left(T\right)={M}_{0}\left(1-B{T}^{n}\right)+ \frac{{C}_{H}}{T-\theta }$, being *M*_*0*_ the spontaneous magnetization at 0 K, *B* the Bloch constant, *n* a parameter which value depends on the size of the particles, *C*_*H*_ a constant in emug^−1^ K and *θ* the Curie–Weiss temperature (negative for antiferromagnets). Table S1 summarizes the obtained parameters of the performed fitting (solid line in Fig. 3). Firstly, the antiferromagnetic nature of the low temperature phase (Curie–Weiss contribution) in the *MIH* treated samples is confirmed through the negative values of *θ* (− 19 K and − 13 K for 20-MNP and 100-MNP *AC*-treated samples, respectively). Regarding the Bloch contribution, similar values of the characteristic parameters (*B* and *n*) have been reported in magnetite within the nanoscale regime^24^. The slight decrease in *B* after the *MIH* procedure would be indicative of an increase of the magnetic order in the magnetite phase. In fact, this effect can also be detected in the evolution of the *ZFC*–*FC* curves (see inset of Fig. 3), where the magnetic ordering can be clearly visualized by the detection of the characteristic Verwey transition at *T*_*V*_ ≈ 120 K^25^. It is reported that the Verwey transition at the nanoscale depends on different factors, being stoichiometry, particularly the cation distribution in the spinel structure (Fe^2+^ and Fe^3+^ in the octahedral sites) the main factor^26^. The ordering of the spinel phase as a consequence of the MNPs self-heating in the presence of a reducing media as fructose, would give rise the observation of the Verwey transition in the *ZFC–FC* magnetization curves. Thus, the performed *MIH* procedure would give rise to a small fraction of antiferromagnetic phase on the MNPs surface simultaneously to the changes within the spinel structure linked to an increase of stoichiometry of the magnetite phase.

**Figure 4:**
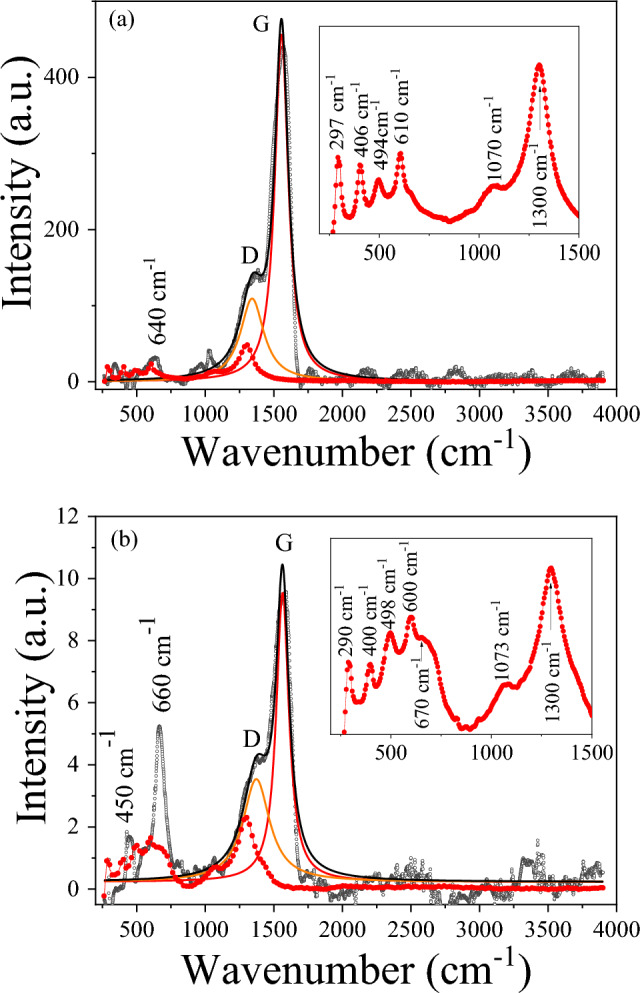
Raman spectra of (**a**) 20-MNPs and (**b**) 100-MNPs: (red filled circle) initial MNPs and (o) *AC*-treated samples. The solid lines represent the Lorentz deconvolution of the peaks in the treated samples. Inset: Enlargement of the low wavenumber region for the initial MNPs.

With respect to the carbon phase, the order state of carbonaceous matrix formed through the *MIH* procedure was studied through Raman spectroscopy. Figure 4 shows the Raman spectra of the *MIH* treated samples, where the Raman spectrum of the initial MNPs is also included as an inset for comparison. For the initial MNPs, bands in the region *ω* < 700 cm^−1^ are detected and attributed to iron oxides (see inset in Fig. 4)^27^. Specifically, the reported *E*_*g*_ modes at 293, 299, 412 and 613 cm^−1^ and *A*_1*g*_ mode at 498 cm^−1^_,_ and the intense peak at 1320 cm^−1^ ascribed to a two-magnon scattering for hematite α-Fe_2_O_3_^28^ can be deduced. However, the coexistence of magnetite (hump around 670 cm^−1^) and other oxides or hydroxides cannot be completely excluded (i.e. maghemite γ-Fe_3_O_2_ at 500 cm^−1^ or hydroxides around 1100 cm^−1^). The characteristic modes for magnetite (*T*_2*g*_ at 300 and 540 cm^−1^, and *A*_1*g*_ at 670 cm^−1^) are not clearly visualized in these initial MNPs. Notice that the occurrence of antiferromagnetic phases such as α-Fe_2_O_3_ (i.e. Morin transition)^29^ is not detected through the magnetic characterization. Thus, it should be concluded that the hematite would be mainly formed during the Raman characterization as a consequence of the oxidation of the magnetite due to the laser irradiation^27,28^. It should be noticed that after the *MIH* procedure, most of the bands attributed to hematite disappear, being the strong band of magnetite around 660 cm^−1^ clearly visible in both samples. However, the occurrence of a band at 450 cm^−1^, especially visible for the 100-MNPs, would not exclude the occurrence of wüstite (FeO). The differences in the carbon coating after the *MIH* procedure in both systems are clearly visible through the Raman spectra (i.e. relative carbon fraction). While for the 20-MNPs it is difficult to distinguish the magnetite bands within the experimental spectrum background, being the carbon signal the main contribution, they are clearly detected for 100-MNPs. This result confirm the lower carbon fraction for these nanoparticles with lower heating capacity, as TEM and the magnetic characterization reflect.

With the respect to the Raman spectra of the *MIH* treated samples, the characteristic *G* and *D* bands of the ordered and disorder carbon, respectively, are clearly detected in the first order region (1100–1800 cm^−1^)^21^. No overtones appear in the second order region (2200–3400 cm^−1^), confirming a disordered (amorphous) state of the carbon present in the samples.

Table 1 summarizes the fitted parameters of the *G* and *D* bands employing a Lorentz peak deconvolution: *ω*_*D*_, *ω*_*G*,_ *FWHM* (full width at half maximum), and the relative intensities of the peaks (*I*_*D*_/*I*_*G*_). Low *I*_*D*_/*I*_*G*_ values (*I*_*D*_/*I*_*G*_ tends to zero in amorphous carbon) together with the decrease of *ω*_*G*_ from 1600 cm^−1^ (graphitic carbon) to around 1510 cm^−1^ (completely disordered carbon)^30,31^, support the conclusion of the amorphous nature of the carbon coating obtained through the performed *MIH* procedure.

**Table 1:** Raman parameters *AC*-treated 20-MNPs and 100-MNPs samples.

Sample	*ω*_*D*_ (cm^−1^)	*ω*_*G*_ (cm^−1^)	*FWHM*_*D*_ (cm^−1^)	*FWHM*_*G*_ (cm^−1^)	*I*_*D*_*/I*_*G*_
Treated 20-MNPs	1342 ± 2	1556.7 ± 0.4	208 ± 7	118 ± 1	0.24
Treated 100-MNPs	1373 ± 5	1564 ± 1	236 ± 14	112 ± 3	0.36

One of the characteristic properties of carbon materials (graphene, nanotubes, graphite and carbon black) is their high electrical conductivity^32^. Taking this property into account, electrical resistance measurements were performed to discern between the different carbon coatings. Accordingly, considering the semiconductor nature of the Fe_3_O_4_ MNPs, an increase in the electrical conductivity would be expected in the carbon coated nanoparticles in comparison with the initial state. In fact, a high electrical resistance (≈ 2 MΩ) is found for the initial nanoparticles, indicating a poor electrical conductivity in the uncoated MNPs. On the other hand, after the *MIH* treatment, a sharp reduction in the electrical resistance (≈ 2 Ω) is detected in both treated samples associated to the formation of carbon coating.

To confirm the formation of the carbon matrix of high electrical conductivity under the performed *MIH* treatment, two additional samples were characterized: initial fructose powder and a sample prepared by mixing 200 mg of the initial 100-MNPs sample with 400 mg of fructose, submitted to an equivalent annealing treatment in a conventional oven at 200 °C (2 h). In both cases, the electrical resistance was higher than 200 MΩ indicating the electrical isolating nature of the samples.

Therefore, it can be concluded that electrically conductive carbon coating is obtained through *MIH* treatment as a consequence of the fructose degradation, while equivalent conventional thermal treatments in an oven are not able to provide a similar microstructure. In fact, the achievement of higher local temperatures at local scale on the nanoparticle surface under the *MIH* experiments would give rise to the sugar degradation and justify the differences with the performed conventional annealing.

The multifunctional features of the synthesised nanostructures (magnetic carbon nanocomposites) enable their application in different technological fields, such as, conductive 3D printing fillers, *RF* absorption components and environmental remediation. As an example, the optimum nanocomposite with highest carbon fraction (*AC-*treated 20 MNP) was employed in Cr (VI) removal tests in aqueous media.

**Figure 5:**
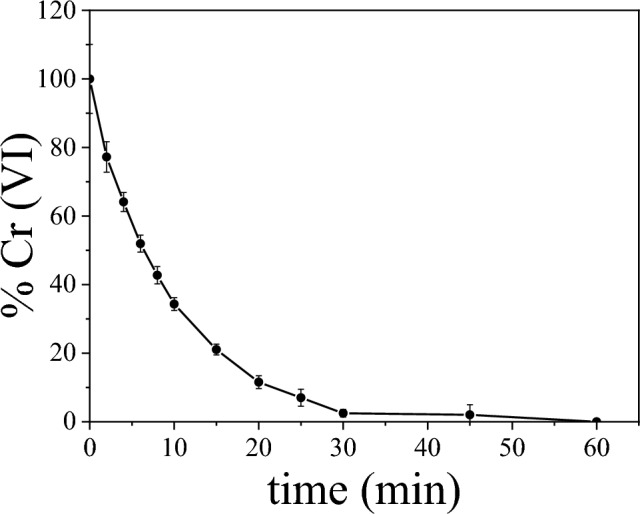
Cr adsorption (%Cr(VI)) in the presence of the *AC*-treated 20-MNP nanocomposite.

Figure 5 displays the Cr adsorption graph (evolution of % Cr (VI) as a function of the contact time) for the selected nanocomposite. The results show a good reproducibility (mean value of 3 tests with the associated error bar). As can be seen, the nanocomposite is able to almost fully absorb the Cr anions in the aqueous solution after 30 min.

The adsorption kinetics of the process were analysed employing two kinetic models widely reported in the literature to characterize the adsorption process in carbon based nanostructures^33^:

1 $${\text{Pseudo-first\,order:}}\;{\text{ln}}(q_{e} - q_{t} ) = {\text{ln}}q_{e} - k_{1} t$$

2 $${\text{Pseudo - second}}\;{\text{order:}}\;\frac{t}{{q_{t} }} = \frac{1}{{k_{2} q_{e}^{2} }} + \frac{t}{{q_{e} }}$$

with *q*_*t*_ and *q*_*e*_ are the amount of pollutant adsorbed (mg_pollutant_/g_adsorbent_) at time* t* (min) and equilibrium, respectively; *k*_*1*_ is the constant for the pseudo-first-order model (min^−1^) and *k*_*2*_ the constant for the pseudo-second-order model (g/(mg·min)). While the pseudo-first-order is correlated with the adsorption process which occurs through diffusion across the interface mainly at first stages, the pseudo-second-order model predicts the behavior over the whole range of adsorption, based on chemical adsorption.

Regarding adsorption isotherms, Langmuir and Freudlich models are usually employed to characterize the adsorption process^33^:

3 $${\text{Langmuir}}\;{\text{model:}}\;\frac{{c_{e} }}{{q_{e} }} = \frac{{C_{e} }}{{q_{{max}} }} + \frac{1}{{q_{{max}} K_{L} }}$$

4 $${\text{Freundlich}}\;{\text{model:}}\;{\text{ln}}\,q_{e} = lnK_{F} + \frac{{lnC_{e} }}{n}$$

where *q*_*e*_ (mg_pollutan_t/g_adsorbent_) corresponds to the amount of the Cr (VI) adsorbed at equilibrium time, *q*_*max*_ (mg_pollutant_/g_adsorbent_) is the maximum adsorption capacity, *C*_*e*_ (mg_pollutant_/L_solution_) the Cr (VI) concentration at the equilibrium, *K*_*L*_ (L/mg) is the Langmuir constant, and *K*_*F*_ (mg/g·(L/mg)^1/n^) and *n* are Freundlich constants. Additionally, a dimensionless constant *R*_*L*_ is employed to explain the adsorption characteristics of the Langmuir isotherm, being: ${R}_{L}=\frac{1}{1+{K}_{L}{C}_{e}}$. For *R*_*L*_ values in the 0–1 range, the adsorption can be considered favorable; *R*_*L*_ = 0 is irreversible; *R*_*L*_ > 1 is unfavorable and *R*_*L*_ = 1 indicates a linear adsorption. In this case, the Langmuir model assumes a homogeneous surface (monolayer process) with no lateral interaction between adsorbed molecules, and reversible adsorption. Freundlich model is based on a heterogeneous surface, multilayer and reversible adsorption.

Table 2 summarizes the adsorption fitting parameters employing the proposed models. Pseudo-second-order and Langmuir models describe slightly better the adsorption kinetics and adsorption isotherms, respectively (highest values of the correlation coefficient *R*^*2*^). Figure S3 shows the corresponding fitting graphs for both models.

**Table 2:** Adsorption fitting parameters employing the different proposed models.

Pseudo-first-order model			Pseudo-second-order model			
*q*_*e*_ (mg/g)	*k*_*1*_ (min^−1^)	*R*^*2*^	*q*_*e*_ (mg/g)	*k*_*2*_ (g/mg min)	*R*^*2*^	
0.417	0.096	0.950	0.244	0.55	0.995	

Langmuir model				Freundlich model		
*q*_*max*_ (mg/g)	*K*_*L*_ (L/mg)	*R*_*L*_	*R*^*2*^	*K*_*F*_ (L/g*)*	*n*	*R*^*2*^
18.6	0.132	0.993	0.991	5.51	3.72	0.945

Therefore, it can be concluded that the chemical adsorption would dominate the process with homogeneous surface adsorption (monolayer process). It should be noted that although the synthesized adsorbent displays reduced values of *q*_*e*_ and *q*_*max*_ compared to other carbon mesoporous adsorbents, *k*_*2*_ and *K*_*L*_ are comparable with the reported constants in other carbon nanostructures^34–36^. Nevertheless, the magnetic response of the nanocomposite enhances its functionality, allowing the magnetic separation from the aqueous media and facilitating its recovery and recyclability.

## Conclusions

A novel carbon coating procedure of Fe_3_O_4_ magnetic nanoparticles (MNPs) is described employing Magnetic Induction Heating (*MIH*). Two sets of MNPs, 20-MNP and 100 MNP, were employed and the differences in the heating capacity under *AC* magnetic field of 305 kHz was analysed as a function of the mean nanoparticle size. Thus, higher heating efficiency is found for the smaller nanoparticles (20-MNP) ascribed to their monodomain magnetic nature in opposite to the larger dimensions of the 100-MNPs. The increase in temperature linked to the *AC* magnetization of the MNPs is able to decompose the fructose when it is mixed with the nanoparticles. No significant changes in the structural and magnetic properties of the MNPs are found after the *MIH* treatment. As a consequence of the thermal decomposition of fructose, carbon based coatings are obtained (disordered carbon matrix), whose relative percentage compared to the fraction of MNPs increases for smaller nanoparticles with greater heating capacity (20-MNP). Nanocomposites with high electrical conductivity are obtained under *MIH* enabling the design of multifunctional nanocomposites with application in different technological fields. As an example, their use as nanoadsorbents for the removal of Cr (VI) in aqueous solutions is demonstrated.

## Methods

Iron (II, III) oxide, nanopowder with grain sizes around 20 and 100 nm were purchased from SIGMA-ALDRICH (Aldrich prod. Num 637106). The magnetic nanoparticles (MNPs) were synthesized through thermal decomposition synthesis of iron acetylacetonate (Fe(acac)_3_). Prior to the carbon nanocomposite synthesis, the induction heating capacity of the magnetic nanoparticles and the MNPs mechanically mixed with fructose in powder form (1:2 weight ratio) were characterized employing a commercial G3 D5 series Multi-mode 3000W Drive from nanoscale Biomagnetics setup (amplitude and frequency of *AC* magnetic field 300 Oe and 305 kHz, respectively). Comparatively, Thermogravimetric (TGA) Analysis, (HI-RES 2950 TA Instruments) employing a heating rate of 10 °C/min under nitrogen atmosphere was employed to analyze the decomposition process in the sugar (fructose) as a function of temperature. For the synthesis of the Fe_3_O_4_@C nanocomposites 200 mg of the MNPs were mechanically mixed with fructose in powder form (1:2 weight ratio) employing a mortar-pestle and then placed in the beaker and introduced into the coil that generated the *RF*(*AC*) magnetic field (305 kHz). The amplitude of the magnetic field was adjusted to control the temperature of the mixture to reach 200 °C at a constant heating rate (≈ 25 °C/min), keeping this temperature for 2 h. Finally, the obtained powder was washed several times with deionized water and the coated magnetic nanoparticles were collected with a magnet. The magnetically separated sample was dried at 60 °C in an oven overnight to eliminate the adsorbed water.

The structural analysis of the samples (i.e. initial MNPs and *AC*-treated MNPs) were performed through X-ray powder diffractometry, XRD, (Bruker D8 advance) with monochromated Cu Kα1 radiation (λ = 1.54056 Å), using Rietveld method and the Fullprof program in the analysis of the spectra^37^. Transmission Electron Microscopy (HRTEM) and Scanning Transmission Electron microscopy with a high angle annular dark field detector (STEM-HAADF) analysis using a FEI Tecnai Field Emission Gun operated at 300 kV, enabled the comparative analysis of the MNPs changes upon the *AC-*magnetic heating treatments and the carbon coating morphology. Raman spectroscopy (Jasco NRS-3100 dispersive Raman spectrophotometer using a 532 nm laser (7 mW) and a 600 line grating covering the range 260–3900 cm^−1^) was used to analyze the carbon order state in the annealed sample. Powder samples, initial MNPs and *AC*-treated MNPs, without further preparation were exposed 0.1 s per scan and at least 500 scans were accumulated in order to get a good signal to noise ratio. A SQUID magnetometer (Quantum Design MPMS XL7) was employed to magnetically characterize the samples. To confirm the graphitic carbon coating on the *AC*-treated MNPs, resistance measurements were performed employing a simple two-point measuring technique. Pellets of the initial and treated MNPs were prepared with two electric contacts (copper wires with similar length) and applying 5 Tons of pressure to properly stablish the electric contact (avoiding the use of metallic glues or welding elements).

Finally, Cr (VI) absorption test were performed employing a UV–Vis spectrophotometer (UV-16, LAN OPTICS) with a wavelength range from 190 to 1100 nm. The experimental procedure is described in^30^. In brief, a colour reagent (1,5-diphenylcarbohydrazide) was employed since aqueous Cr (VI) solutions have not absorption in the UV–Vis range. Calibration curves were initially performed to properly measure the Cr (VI) concentration in the aqueous solutions. For each test (pH = 6), 25 mL of Cr (VI) water solution (1 mg/L Cr (VI)) with 25 mg of the adsorbent was prepared. Then, the solutions were mechanically stirred and aliquots of 1 mL were collected at different times, the adsorbent was separated by a hand-held magnet, and the solution was filtered by a 0.22 μm syringe filter. The adsorption kinetics were analysed employing pseudo-first-order and pseudo-second-order models. For Cr (VI) adsorption isotherms, aqueous solutions with different Cr (VI) concentrations (from 0.2 to 100 mg L^−1^) were prepared with the same amount of adsorbent (1 mg mL^−1^). Langmuir and Freundlich models were employed to evaluate the adsorption isotherms of the studied samples.

## Supplementary Information

Supplementary Information.

## Data Availability

The datasets generated during and/or analysed during the current study are available from the corresponding author on reasonable request.
