# Author Correction: Magnetic carbon Fe_3_O_4_ nanocomposites synthesized via Magnetic Induction Heating

**DOI:** 10.1038/s41598-023-35598-3

**Published:** 2023-06-01

**Authors:** L. Cervera‑Gabalda, C. Gómez‑Polo

**Affiliations:** 1 Departamento de Ciencias, Universidad Pública de Navarra, Campus de Arrosadia, 31006 Pamplona, Spain; 2 Institute for Advanced Materials and Mathematics (INAMAT2), Universidad Pública de Navarra, Campus de Arrosadia, 31006 Pamplona, Spain

Correction to: *Scientific Reports* 10.1038/s41598-023-34387-2, published online 04 May 2023

The Acknowledgements section in the original version of this Article contained an error in the funding agency.

“The research was funded by MCIN/AEI/https://doi.org/10.13039/501100011033, Grant PID2020-116321RB-C21.”

now reads:

“The research was funded by MCIN/AEI/10.13039/501100011033, Grant PID2020-116321RB-C21.”

The original Article has been corrected.

