# Supplementary material for: Magnetic carbon Fe3O4 nanocomposites synthesized via Magnetic Induction Heating
Source: Sci Rep. 2023 May 4;13:7244. doi: 10.1038/s41598-023-34387-2 (PMC10160050; doi:10.1038/s41598-023-34387-2)
Supplement: Supplementary file 1 — Supplementary Information. [file 41598_2023_34387_MOESM1_ESM.pdf]

SUPLEMENTARY INFORMATION

Magnetic carbon Fe_3O_4 nanocomposites synthesized via Magnetic Induction Heating

L. Cervera-Gabalda [1,2] and C. Gómez-Polo[1,2*]

[1]Departamento de Ciencias, Universidad Pública de Navarra, Campus de Arrosadia, 31006 Pamplona, Spain.

[2]Institute for Advanced Materials and Mathematics (INAMAT[2]), Universidad Pública de Navarra, Campus de Arrosadia, 31006, Pamplona, Spain.

*gpolo@unavarra.es

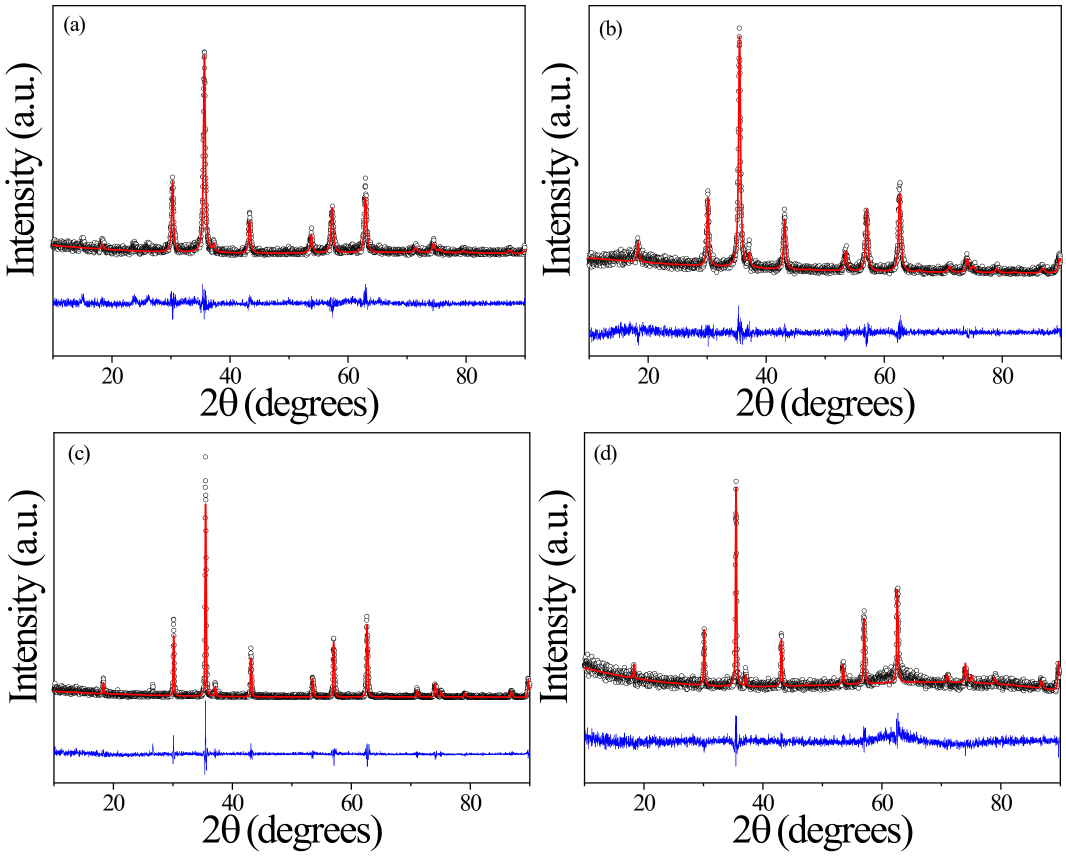

Figure S1. XRD patterns and Rietveld refinement of 20-MNP, (a) initial and (b) *AC*-treated; and 100-NMP (c) initial and (d) *AC*-treated. (o) Experimental, (—) calculated intensities and (—) difference between both intensities.

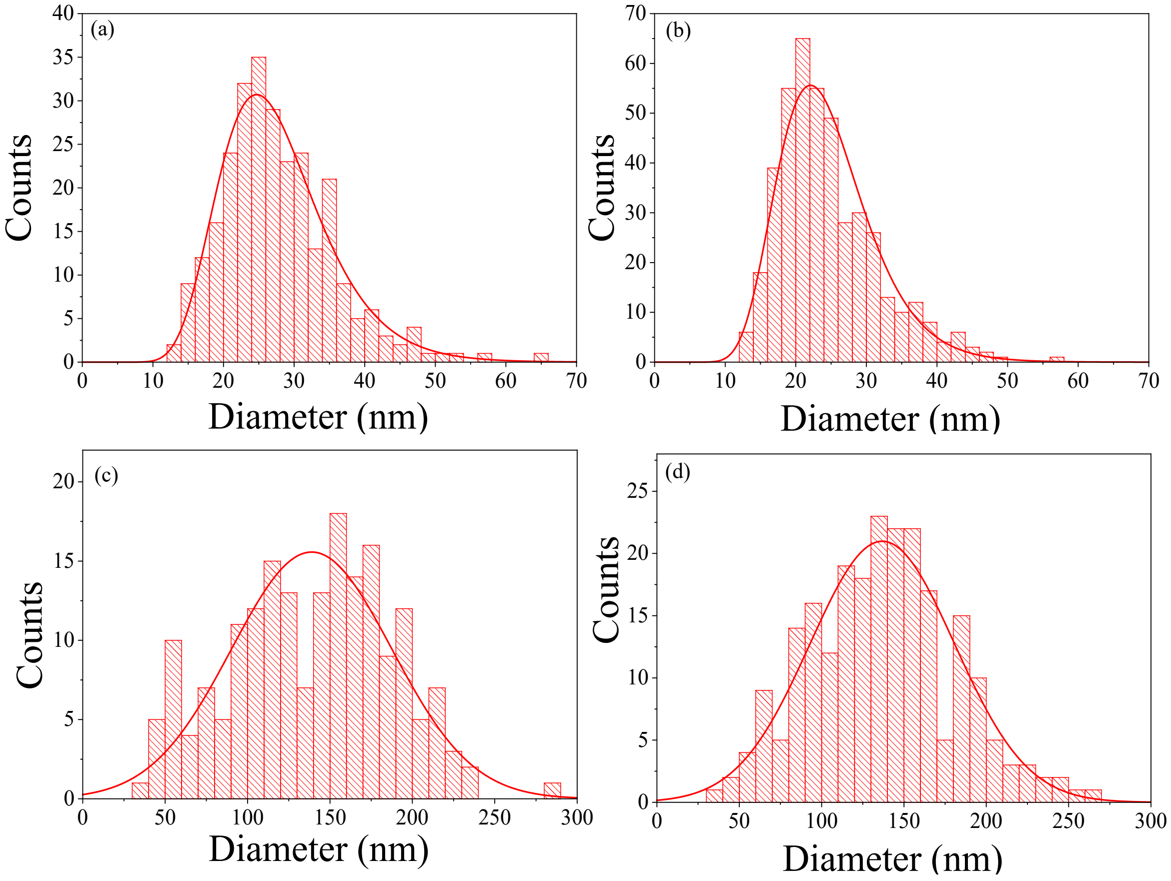

Figure S2. TEM histograms of the nanoparticle for 20-MNP, (a) initial and (b) *AC*-treated; and 100-NMP (c) initial and (d) *AC*-treated.

Table S1: Bloch law fitting parameters including the Curie-Weiss contribution.

sample	M_0 (emu/g)	*B* (K^{-n})x10^{-8}	*n*	C_H (emu/g K)	*θ* (K)
20-MNP	85.37±0.01	8.5±0.6	2.06±0.01	--	--
Treated 20-MNP	45.40±0.04	1.4±0.8	2.7±0.1	63±3	-18.6±0.9
100-MNP	88.17±0.01	7.0±1.0	2.43±0.03	--	--
Treated 100-MNP	80.95±0.03	1.3±0.3	1.90±0.02	18±2	-13±2

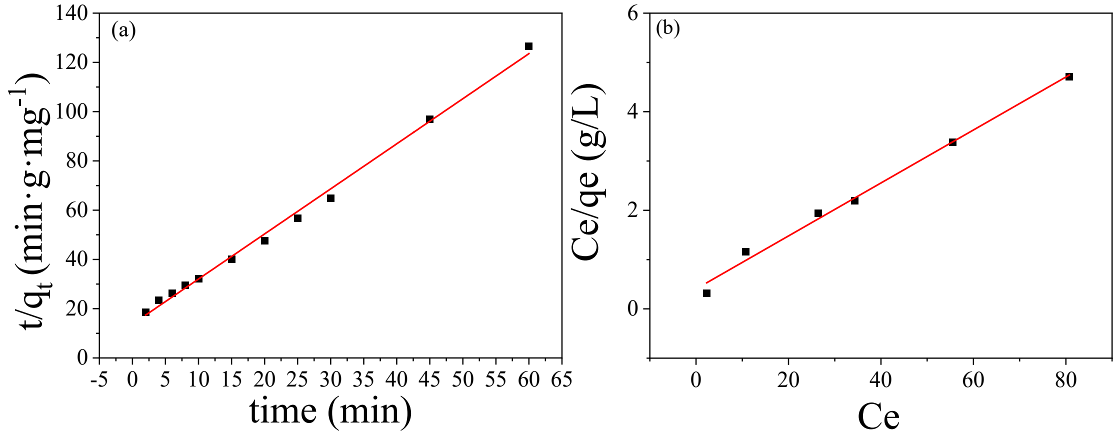

Figure S3. (a) Adsorption kinetics (*Pseudo-second-order model*) of Cr(VI) and (b) adsorption isotherms (*Langmuir model*) employing 20-MNP *AC*-treated sample.
